# Electrochemically Fabricated Surface-Mesostructured CuNi Bimetallic Catalysts for Hydrogen Production in Alkaline Media

**DOI:** 10.3390/nano12010118

**Published:** 2021-12-30

**Authors:** Jingyuan Bai, Jin Zhang, Konrad Eiler, Zhou Yang, Longyi Fan, Dalong Yang, Meilin Zhang, Yupu Hou, Renguo Guan, Jordi Sort, Eva Pellicer

**Affiliations:** 1 School of Materials Science and Engineering, Northeastern University, Shenyang 110819, China; baekkyungwon@163.com (J.B.); zhangmeilin9696@163.com (M.Z.); 2 Engineering Research Center of Continuous Extrusion, Ministry of Education, Dalian Jiaotong University, Dalian 116028, China; jinzhang@djtu.edu.cn (J.Z.); yangzhou5959@163.com (Z.Y.); fanlongyi98@163.com (L.F.); ydl@djtu.edu.cn (D.Y.); houyupu1999@163.com (Y.H.); 3 Center of Advanced Lubrication and Seal Materials, State Key Laboratory of Solidification Processing, Northwestern Polytechnical University, Xi’an 710072, China; 4 Departament de Física, Facultat de Ciències, Universitat Autònoma de Barcelona, E-08193 Bellaterra, Cerdanyola del Vallès, Spain; Jordi.Sort@uab.cat; 5 Institució Catalana de Recerca i Estudis Avançats (ICREA), Pg. Lluís Companys 23, E-08010 Barcelona, Spain

**Keywords:** CuNi, micelle-assisted electrodeposition, mesostructured surface, hydrogen evolution reaction

## Abstract

Ni-based bimetallic films with 20 at.% and 45 at.% Cu and mesostructured surfaces were prepared by electrodeposition from an aqueous solution containing micelles of P123 triblock copolymer serving as a structure-directing agent. The pH value of the electrolytic solution had a key effect on both the resulting Cu/Ni ratio and the surface topology. The catalytic activity of the CuNi films toward hydrogen evolution reaction was investigated by cyclic voltammetry (CV) in 1 M KOH electrolyte at room temperature. The Cu_45_Ni_55_ film showed the highest activity (even higher than that of a non-mesostructured pure Ni film), which was attributed to the Ni content at the utmost surface, as demonstrated by CV studies, as well as the presence of a highly corrugated surface.

## 1. Introduction

Bimetallic CuNi materials show significantly different properties compared to monometallic Ni [1,2]. For example, mesostructured CuNi films exhibit intrinsic magnetoelectric effects, in which a drastic reduction in coercivity can be observed under the application of voltage across an electric double layer [3]. The addition of Cu to Ni also promotes the catalytic activity and selectivity of Ni toward a variety of reactions. In carbon dioxide hydrogenation, Cu favors CO_2_ adsorption and CO production, which determines the CO_2_ conversion efficiency [4]. The addition of a small amount of Cu to Ni/Al_2_O_3_ increases the selectivity of 1,3-butadiene hydrogenation toward 1-butene [5]. An enhancement of the hydrogen evolution reaction (HER) of CuNi films with respect to their single counterparts has also been reported [6]. In general, the improvement observed in bimetallic systems tested at HER is due to a variety of reasons: preferential segregation of the most active metal to the surface, changes in the electronic properties, formation of large electrochemically active surface areas, and combinations thereof [7,8,9,10].

An increase in the electrocatalytic activity of CuNi bimetallic materials has been achieved by either modifying their structure and/or surface composition. These features, in turn, are determined by the synthesis parameters [11,12,13]. Wet chemical routes for the preparation of electrocatalysts commonly involve diffusion, adsorption, nucleation, particle growth, and phase transformation. Each step needs to be well understood and controlled to efficiently improve the performance of the resulting heterogeneous catalysts. Interestingly, Cu and Ni possess a similar crystal structure with lattice constants of 3.61 Å and 3.52 Å, respectively, but dissimilar electronic configuration. This is because Cu has a completely filled *3d* band, while Ni has partially filled *3d* orbitals with a high density of states at the Fermi level. As a result, Ni typically shows higher chemical reactivity than Cu. Indeed, surface enrichment in Ni has been shown to increase the catalytic performance of CuNi materials [14]. Yet, such enrichment is not straightforward because Cu has lower surface energy and slightly larger metallic radius; therefore, it is prone to occupy surface sites [15,16]. It is worth noting that the kinetics associated with material preparation plays a key role in determining the final microstructure. For example, a core–shell structure with an Ni-rich surface was obtained by solvated metal atom impregnation [17]. Electrodeposition has proven successful in synthesizing bimetallic CuNi with tunable microstructures and surface composition [18,19,20,21,22]. Concerning electrodeposition, the difference in both the equilibrium potentials and the nucleation rates for Cu and Ni is quite large, enabling the on-demand deposition of porous, multilayered, or fully dense microstructures with Ni-rich surfaces.

The electrosynthesis of films with surface roughness on the macro- and mesoscales has been intensively investigated in the past years as these materials might show increased activity toward HER by virtue of their higher density of active sites. In this context, macroporous bimetallic CuNi films with Ni contents ranging from 15% to 35% were successfully prepared using the hydrogen bubble template-assisted electrodeposition approach [18]. Surprisingly, CuNi films with the lowest Ni content showed the highest specific activity. In HER, the composition of the utmost surface in contact with the electrolyte is crucial as the surface is involved in the very first step of the interaction with water molecules and, furthermore, with the hydrogen gas desorption step [23,24]. The introduction of Cu is thought to enhance the HER activity of Ni by facilitating molecular hydrogen release from traps (by decreasing the hydrogen binding energy) [18]. In continuation to our previous work on macroporous CuNi films [18], films of the same system are herein prepared by aqueous electrodeposition using poly(ethylene oxide)-*block*-poly(propylene oxide)-*block*-poly(ethylene oxide) (PEO–PPO–PEO) triblock copolymer (P123) as a structure-directing agent [25,26,27,28]. The large surface area endowed by the resulting mesostructured surfaces makes the system a suitable candidate as an electrocatalyst for HER in alkaline conditions.

The goal of this paper is to study the HER activity of bimetallic CuNi films prepared by electrodeposition from an aqueous solution containing the P123 triblock copolymer. The electrochemical surface area (ECSA) and surface composition of the electrocatalysts were evaluated by electrochemical means. Furthermore, we demonstrate for this particular alloy that, a higher amount of Ni at the surface leads to better HER performance, thereby confirming that the electrocatalytic activity is well correlated with ECSA and surface composition.

## 2. Materials and Methods

### 2.1. Synthesis of Cu_20_Ni_80_ and Cu_45_Ni_55_ Films

Cu_20_Ni_80_ and Cu_45_Ni_55_ films with mesoscale surface roughness (denoted as ‘meso’) were electrodeposited from a solution containing 0.008 g/mL P123, 0.2 M Ni(OCOCH_3_)_2_∙4H_2_O, 0.02 M CuSO_4_∙5H_2_O, 0.5 M NaOCOCH_3_, 0.2 M boric acid, and 0.4 mg/mL saccharine. The deposition was performed galvanostatically at *j* = −100 mA·cm^−2^ for 150 s at room temperature. The pH of the solution was left at its unadjusted value for the electrodeposition of Cu_20_Ni_80_ (pH = 6.25), while it was brought to 4 with the addition of sulfuric acid for Cu_45_Ni_55_ deposition. Si/Ti (25 nm)/Au (125 nm) substrates (with a working area of 0.25 cm^2^) were used as the cathode. A platinum wire served as the counter electrode, and a double junction Ag|AgCl 3 M KCl electrode (*E* = + 0.210 V versus standard hydrogen electrode (SHE)) was utilized as the reference electrode. N_2_ was bubbled through the solution to get rid of oxygen before each deposition. Following deposition, the films were carefully rinsed with Milli-Q water and placed in isopropanol for 24 h in order to remove P123 remnants. All the chemicals were purchased from Sigma-Aldrich and used without further purification. CuNi films of same composition and pure Ni films, both without mesoscale surface roughness (denoted as ‘plain’ films), were prepared from different electrolytes, as reported elsewhere [29].

### 2.2. Electrocatalytic Activity toward HER

The electrochemical activity of the CuNi films toward HER was investigated in the same setup used for electrodeposition. The Pt counter electrode was in the same compartment as the working and reference electrodes. Both plain and surface-mesostructured films previously deposited on Si/Ti/Au substrates were used as working electrodes. Cyclic voltammetry (CV) curves were recorded in de-aerated 1 M KOH solution by cycling the potential between –1.6 V and +0.2 V at 50 mV·s^−1^ versus Ag|AgCl. The onset potential and current density values of Cu_20_Ni_80_ and Cu_45_Ni_55_ surface-mesostructured films were compared with those of pure Ni, Cu_20_Ni_80_, and Cu_45_Ni_55_ plain films. All the measurements were carried out at 25 °C. The measured potentials vs. Ag|AgCl were converted to the reversible hydrogen electrode (RHE) scale according to

E_RHE_ = E_Ag|AgCl_ + 0.059 pH + E^0^_Ag|AgCl_, (1)

where E_RHE_ is the converted potential vs. RHE, E^0^_Ag|AgCl_ is 0.210 V at 25 °C, and E_Ag|AgCl_ is the experimentally measured potential against the Ag|AgCl reference electrode. *iR* compensation was performed after determination of the instrumentation resistance by electrochemical impedance spectroscopy (EIS), using a potential amplitude of 10 mV and a frequency range between 100 kHz and 10 mHz. Galvanostatic long-term stability tests were performed at −10 mA·cm^−2^ for 24 h.

ECSA values for Cu and Ni were estimated following the procedure described in [14]. Specifically, first the peak areas for Cu_2_O and α-Ni(OH)_2_ formation were gleaned from CV, and then the mass loadings for Ni and Cu were determined. The latter were obtained from the actual mass of the deposited films using a scale with an accuracy of 0.01 mg (Mettler Toledo MS205DU) and considering the relative amounts of Cu and Ni gathered from EDX analyses. Accordingly, ECSA was estimated as

$$\mathrm{ECSA}=\frac{Q_{r}}{m\times C},$$

where *Q_r_* is the charge determined by CV, *m* is the Ni or Cu mass loading, and *C* is the charge of a full monolayer coverage of Cu_2_O (544 μC·cm^−2^) or α-Ni(OH)_2_ (514 μC·cm^−2^). This method of ECSA determination, referred to as ‘Alpha’ [30], was first devised by Machado et al. for bulk Ni [31], and variations of these method have been recently applied to Ni foams [32]. According to Baranova and coworkers [30], the Alpha method provides a precise estimation of the Ni-related ECSA unless the original electrode is made of Ni oxides. Four replicates of each type, namely, Cu_20_Ni_80_ and Cu_45_Ni_55_ surface-mesostructured films, were electrodeposited for element-selective ECSA determination. Values are given as the mean value ± standard deviation.

Finally, the turnover frequency (TOF), which gives the number of H_2_ molecules evolved per second per active site, was determined at −0.25 V vs. RHE following the approach described in [33]. The active sites per real surface area were obtained on the basis of crystallographic considerations of the CuNi unit cell [26].

### 2.3. Structural Characterization

Scanning electron microscopy (SEM) images and energy-dispersive X-ray spectroscopy (EDX) analyses were performed on a Zeiss Merlin microscope operated at 3 kV and 20 kV, respectively. *θ/2θ* X-ray diffraction (XRD) patterns were recorded on a Philips X’Pert diffractometer with a pixel^1D^ detector in the 42–54° 2*θ* range (step size = 0.026°, total time = 1200 s) using Cu *K_α_* radiation (*λ* = 0.154178 nm). Surface adsorption and desorption of N_2_ were studied using a Micromeritics ASAP 2000 particle analyzer. The pore size distribution was calculated by nonlocal density functional theory. Ten replicates of Cu_20_Ni_80_ and Cu_45_Ni_55_ surface-mesostructured films were synthesized and detached from the substrate in order to accumulate a sufficient amount of material. Prior to recording the adsorption-desorption isotherms, the free-standing layers were degassed at 200 °C for 4 h.

## 3. Results and Discussion

**Figure 1:**
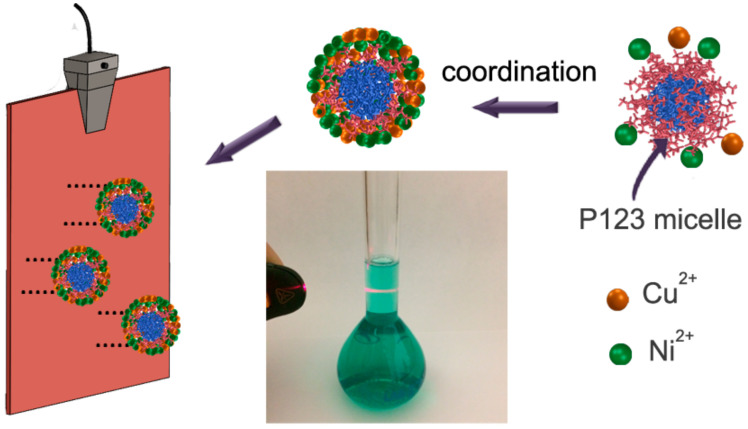
Schematic illustration of the preparation process. The photograph at the center of the sketch demonstrates the occurrence of the Tyndall effect.

In the present study, Cu_20_Ni_80_ and Cu_45_Ni_55_ films with mesostructured surfaces were deposited on an Au surface from an aqueous solution containing the metal salts and the nonionic amphiphilic P123 surfactant. In contrast to the lyotropic liquid crystal (LLC) method, which requires high surfactant concentrations [25], here, a diluted surfactant solution was utilized instead [26]. This approach is referred to as micelle-assisted electrodeposition. In the plating solution, dissolved Cu and Ni ions are coordinated by water molecules forming metal–aqua complexes which adsorb onto the external ethylene oxide (EO) groups of the P123 micelles. During the electrodeposition process, the metal ion species move toward the cathode via coulombic attraction together with the micelles under the applied potential/current density. Hence, the surface of the working electrode acts as a solid–liquid interface, where the metallic films are deposited and patterned by the surfactant–inorganic aggregates. Indeed, the P123 block copolymer has the role of a structure-directing agent (Figure 1). The formation of micelles in solution is key for the development of surfaces with roughness on the mesoscale. The presence of micelles in the electrolyte was proven via the so-called Tyndall effect (Figure 1). Accordingly, the red light pointing to the volumetric flask containing the electrolyte was scattered and reflected by the individual suspension micelles, making the beam visible.

**Figure 2:**
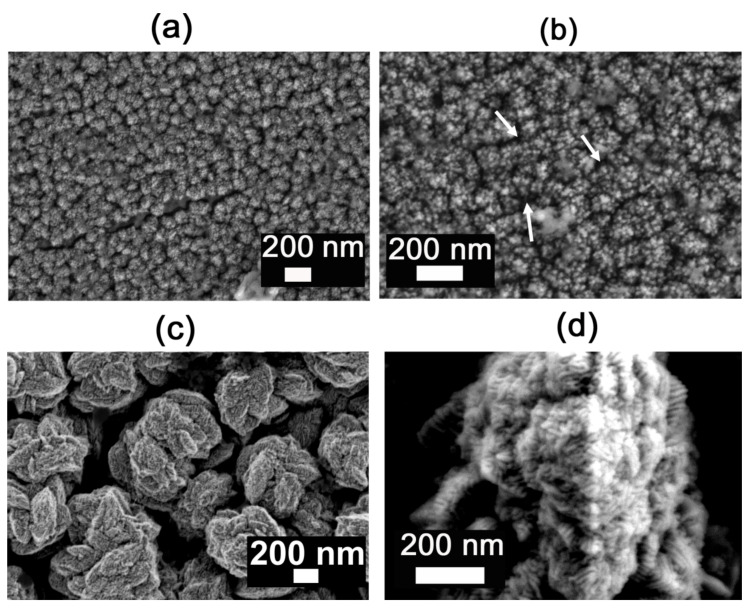
Low- and high-magnification SEM images of surface-mesostructured films with composition (**a**,**b**) Cu_20_Ni_80_ and (**c**,**d**) Cu_45_Ni_55_.

**Figure 3:**
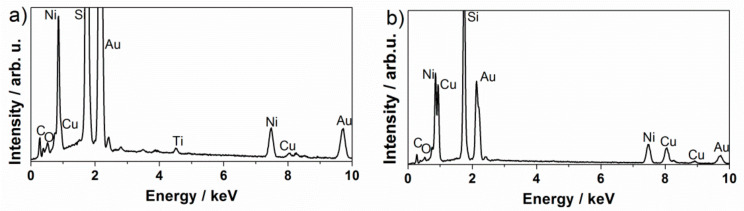
EDX spectra of the surface-mesostructured films with composition (**a**) Cu_20_Ni_80_ and (**b**) Cu_45_Ni_55_.

**Figure 4:**
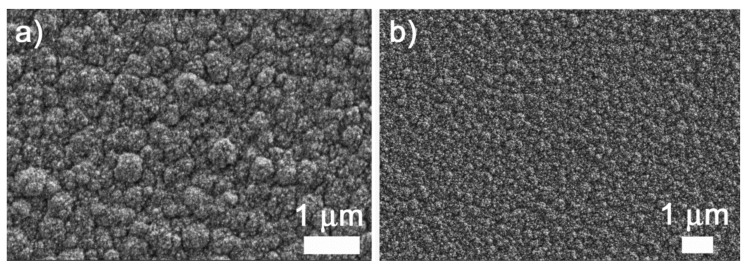
SEM images of CuNi films deposited from the same electrolyte composition at pH values of (**a**) 3 and (**b**) 2.

**Figure 5:**
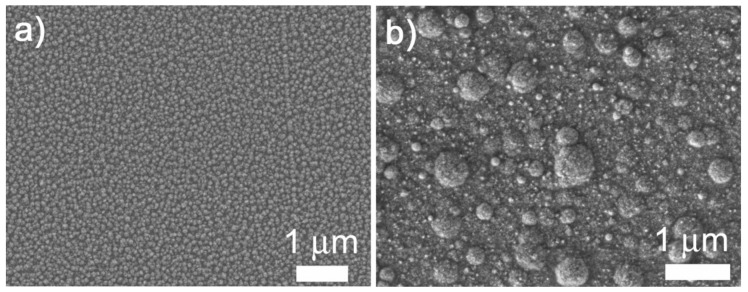
SEM images of films electrodeposited at *j* = –100 mA·cm^−2^ and 150 s from (**a**) the base electrolyte and (**b**) the base electrolyte without Cu ions, at pH = 6.25. Note that the SEM image of (**a**) corresponds to a lower-magnified image of the deposit shown in Figure 2a.

The surface-mesostructured films prepared by micelle-assisted electrodeposition were analyzed by SEM, and the corresponding images are depicted in Figure 2. When the deposition was performed from the electrolyte at pH = 6.25, the resulting CuNi deposits showed globular grains with a fluffy appearance (see Figure 2a). At a higher magnification (Figure 2b), a sort of hierarchical architecture consisting of aggregates of nanoparticles was unveiled. The surface nanovoids result from the interstices left by the aggregation of these nanoparticles (see white arrows in Figure 2b), whose size ranges from 5 to 10 nm. A distinct morphology was observed when deposition was performed at a lower bath pH. In particular, a striated pattern was seen when the pH value of the electrolyte was set to 4 (Figure 2c,d). The observed drastic change in morphology was probably due to an enhanced interaction between metal ions and P123 micelles at pH = 4. Indeed, the morphology is dependent on the type and strength of the interaction (electrostatic, hydrogen bonding, etc.) between the metal ions and surfactant assemblies [34]. In fact, attempts to produce surface-mesostructured CuNi films with lower amounts of Ni by further decreasing the pH of the electrolyte failed because mesoscale roughness did not develop as such. The corresponding EDX spectra (Figure 3) confirm that the films were composed of Cu and Ni with a mean atomic composition of Cu_20_Ni_80_ and Cu_45_Ni_55,_ respectively. As shown in Figure 4, when the pH value was set at 3 or 2, although the resulting films were rough, no signs of mesostructuration were noted. The presence of Cu ions in the electrolyte was key in promoting the occurrence of surfaces with mesoscale features. Specifically, when the deposition was carried out in the absence of the Cu salt, while keeping the remaining conditions unchanged, the resulting pure Ni film showed a more compact, bumpy, fine-grained morphology (Figure 5). This observation is in good accordance with the results previously reported by our group, i.e., the presence of Cu ions in the electrolyte, and their discharge is somewhat related to the successful formation of mesostructured films, at least for the current electrolyte formulation and deposition conditions used here [26].

**Figure 6:**
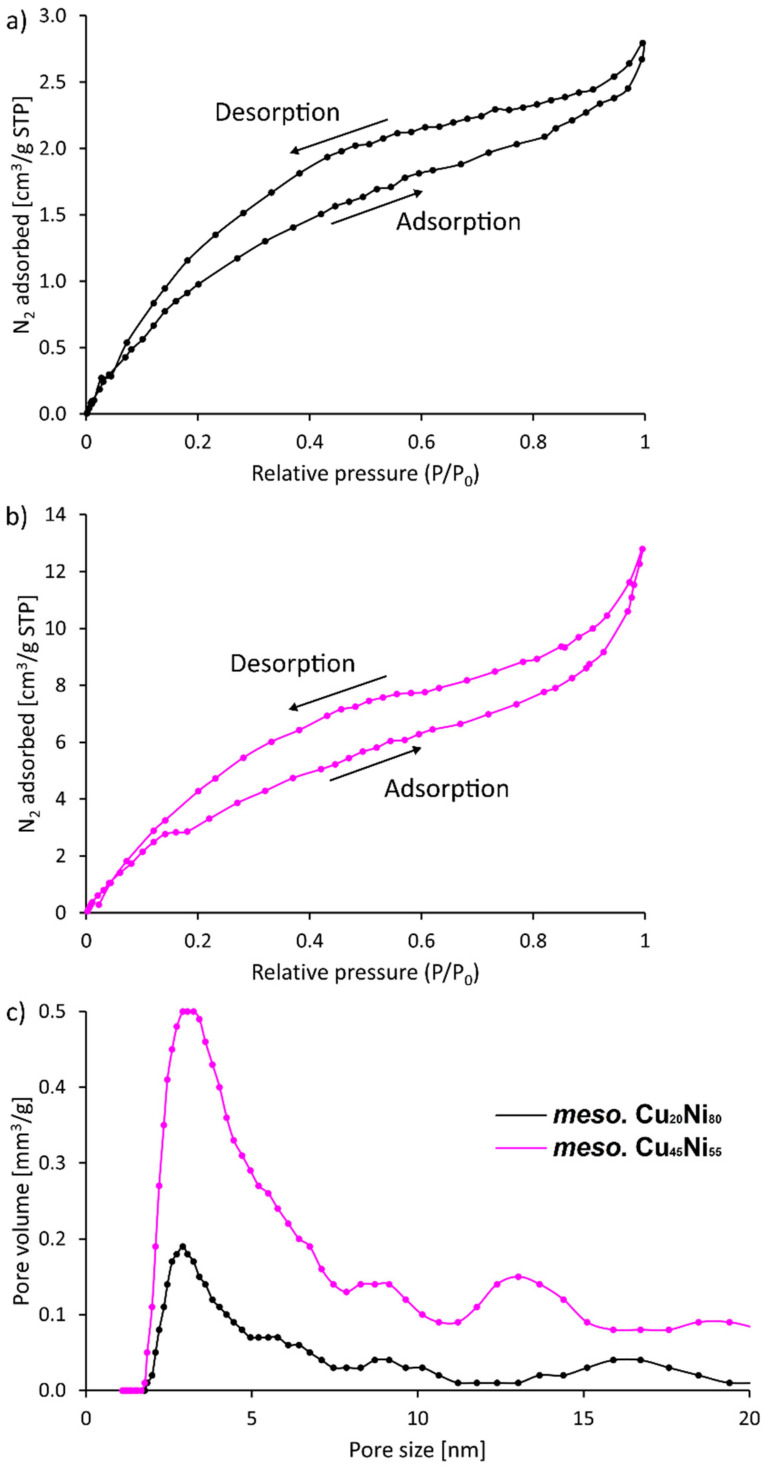
N_2_ isotherms for (**a**) Cu_20_Ni_80_ and (**b**) Cu_45_Ni_55_ surface-mesostructured films; (**c**) resulting pore size distribution for both compositions.

N_2_ adsorption–desorption curves were taken to gain further information on the roughness and porosity of the obtained films. A hysteresis was observed within the whole range of P/P_0_ values (Figure 6). However, the fact that the isotherm branches did not swing sharply upward near P/P_0_ = 0.9 indicate that mesopores were not uniformly present inside the material. Indeed, the resulting pore size distributions were ill-defined for both compositions (Figure 6c). Hence, the results point toward the formation of rough surfaces with mesoscale features, as previously seen by SEM.

**Figure 7:**
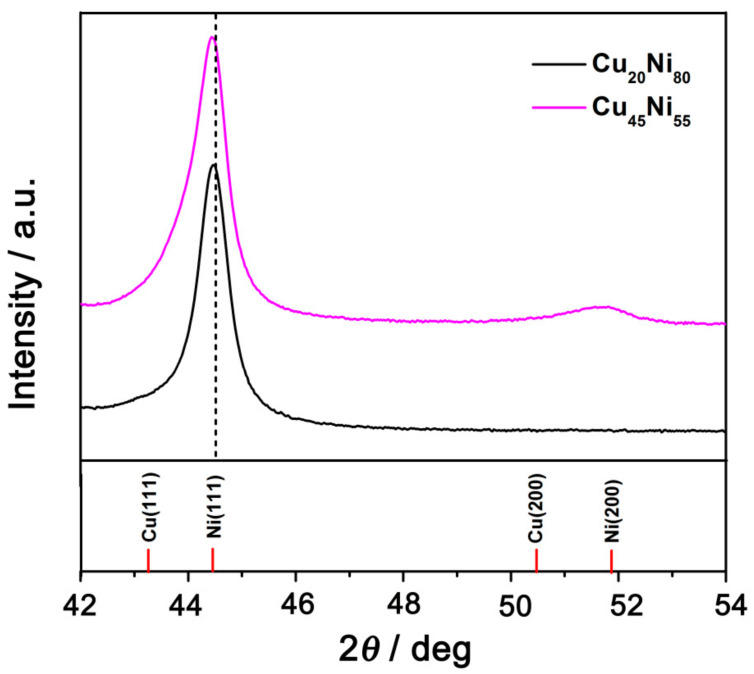
Zoomed-in view of the XRD patterns of surface-mesostructured Cu_20_Ni_80_ and Cu_45_Ni_55_ films.

Figure 7 shows the XRD patterns of Cu_20_Ni_80_ and Cu_45_Ni_55_ surface-mesostructured films between 42° and 54°. In this region, two peaks at ca. 44° and one at 51–52° were observed, located between the (111) and (200) peaks of face-centered cubic (fcc) crystal structures of Ni and Cu. This indicates that the samples crystallized in the fcc structure (space group Fm3m). The position of the (111) reflection for Cu_20_Ni_80_ shifted toward lower angles with respect to that of Cu_45_Ni_55_, from 44.8° to 44.3° (ca. 0.5°), due to the increase in the Cu content. For both Cu_45_Ni_55_ and Cu_20_Ni_80_ samples, although peak splitting was not observed, the asymmetric broadening of the (111) peak gave a clear indication for the coexistence of Cu-rich and Ni-rich phases (i.e., two fcc solid solutions with slightly different cell parameters). Moreover, the (200) fcc peak, which should appear at around 2*θ =* 51–52°, was not detected for Cu_20_Ni_80_ and had a relatively low intensity for Cu_45_Ni_55_, suggesting a pronounced crystallographic texture along the (111) direction [26].

**Figure 8:**
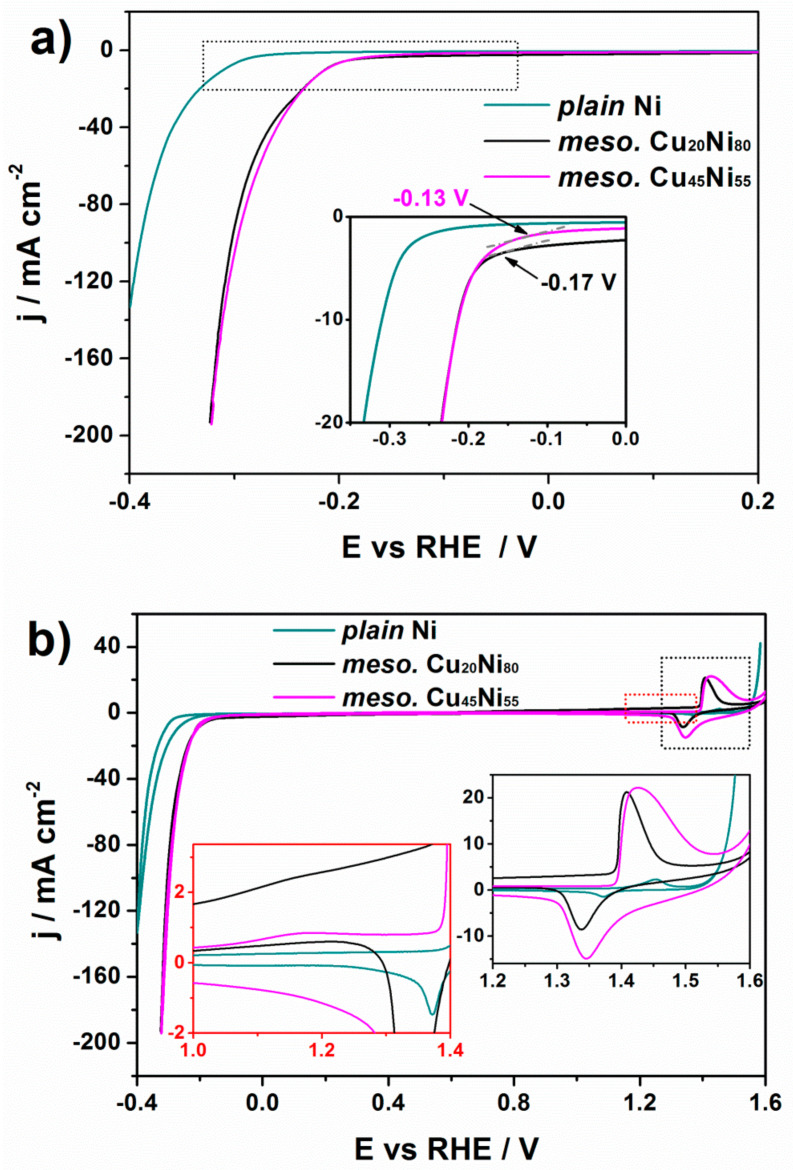
(**a**) Polarization and (**b**) CV curves recorded in 1 M KOH electrolyte for Cu_20_Ni_80_ and Cu_45_Ni_55_ surface-mesostructured films. The response of a plain pure Ni film is shown for comparison. The inset in (**a**) and right inset in (**b**) show zoomed-in parts of the curves enclosed within the black dotted rectangle. The left inset in (**b**) shows a zoomed-in view of the region enclosed in the red dotted rectangle.

**Figure 9:**
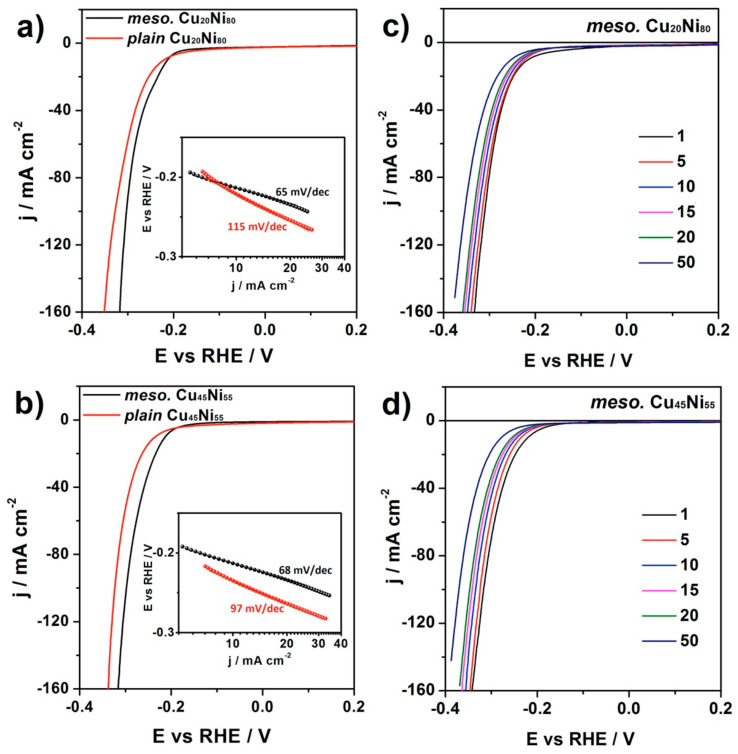
Cathodic-going linear sweep voltammetry curves for comparison between plain and surface-mesostructured (**a**) Cu_20_Ni_80_ and (**b**) Cu_45_Ni_55__,_ and the variation in HER current density as a function of the number of scans for (**c**) Cu_20_Ni_80_ and (**d**) Cu_45_Ni_55_. All curves were recorded in recorded in 1 M KOH at a scan rate of 50 mV·s^−^^1^ and 298 K. The insets in (**a**) and (**b**) show the Tafel slopes.

The catalytic activity toward HER of the Cu_20_Ni_80_ and Cu_45_Ni_55_ surface-mesostructured films was studied by recording polarization curves in 1 M KOH solution at a sweeping rate of 50 mV·s^−1^ at room temperature (Figure 8a). Note that current density is given per geometric area of the film exposed to the electrolyte. For comparison purposes, the behavior of a plain Ni film was used as a reference material. Since the onset potential can be determined from the deviation of the potential in the linear region [35], as indicated by an arrow in the inset of Figure 8a, one can conclude that the surface-mesostructured Cu_45_Ni_55_ showed lower onset potentials than those of plain Ni and surface-mesostructured Cu_20_Ni_80_ films. Polarization curves of plain Cu_20_Ni_80_ and Cu_45_Ni_55_ were also measured, as shown in Figure 9. Surface-mesostructured samples exhibited lower onset potentials, higher geometric current densities, and lower Tafel slopes than their plain counterparts (see Table 1). The enhanced HER performance of the mesostructured films was mainly due to their larger surface areas and the preferential segregation of Ni at the surface, as explained below.

In order to gain information regarding surface composition of the films, a CV study was carried out in 1 M KOH at a scan rate of 50 mV·s^−1^ (Figure 8b). For both Cu_20_Ni_80_ and Cu_45_Ni_55_ surface-mesostructured films, the small peak placed at approximately +1.16 V could be ascribed to the formation of Cu_2_O (Cu + OH^−^ → Cu(OH) + e^−^), as shown in the left inset in Figure 8b. Likewise, from the right inset in Figure 8b, the anodic peak within the 1.39–1.57 V region could be attributed to the oxidation of Ni (0) to Ni (2+) to deliver α-Ni(OH)_2_. Meanwhile, the cathodic peak at around 1.29–1.44 V was due to the reduction of α-Ni(OH)_2_ to Ni (0). These two peaks could be detected for the three samples; in particular, they remained despite the presence of Cu in the CuNi samples. This indicates that the oxidation of Cu and Ni from surface-mesostructured CuNi films took place independently, without involving the formation of mixed oxides. Therefore, in a first approximation, the ECSA values for Ni and Cu can be derived from the charge related to the formation of α-Ni(OH)_2_ and Cu_2_O monolayers, and they are given with respect to the electrodeposited mass [14]. The charge under a monolayer formation of α-Ni(OH)_2_ [31,36] is 514 μC·cm^−^^2^, while that of Cu_2_O [37] is 544 μC·cm^−^^2^. Considering these, the resulting ECSA values for Ni and Cu are given in Table 1. As the Cu content increased in the film, the Cu-related ECSA increased from 0.3 m^2^·g^−1^ to 0.4 m^2^·g^−1^. More interestingly, the Ni-related ECSA decreased with the Ni content, a result which might seem counterintuitive and suggests that the number of active Ni sites at the surface can be increased by alloying Ni with Cu. The same observation was made by Jang and Kim [14], who also demonstrated that Ni segregates at the surface with addition of Cu to the material. Overall, the surface-mesostructured Cu_45_Ni_55_ film showed higher Ni-related ECSA, and, since Ni was more intrinsically active toward HER than Cu according to the Volcano plot [38], this resulted in an increased HER performance. Table 1 also lists the overpotential at −10 mA·cm^−2^ (η_10_) and the TOF values of the CuNi films with mesoscale roughness. Interestingly, the former was lower compared to their plain counterparts.

In order to examine the catalytic durability of the materials in the alkaline electrolyte within a short time scale, Cu_20_Ni_80_ and Cu_45_Ni_55_ surface-mesostructured films were subjected to 50 cycles in 1 M KOH electrolyte within the potential range from 0.2 V to −0.5 V vs. RHE. As shown in Figure 9c,d, the HER current density of the CuNi films showed the highest value at the first cycle and then decreased in subsequent cycles, indicating a faint loss of activity. The shift in the onset potential toward more negative values after 50 cycles was larger for Cu_45_Ni_55_ compared to Cu_20_Ni_80_. This was probably due to the larger Ni-related ECSA of Cu_45_Ni_55_; thus, the more vigorous hydrogen evolution required longer time to reach the equilibrium state of surface tension and buoyancy force.

**Figure 10:**
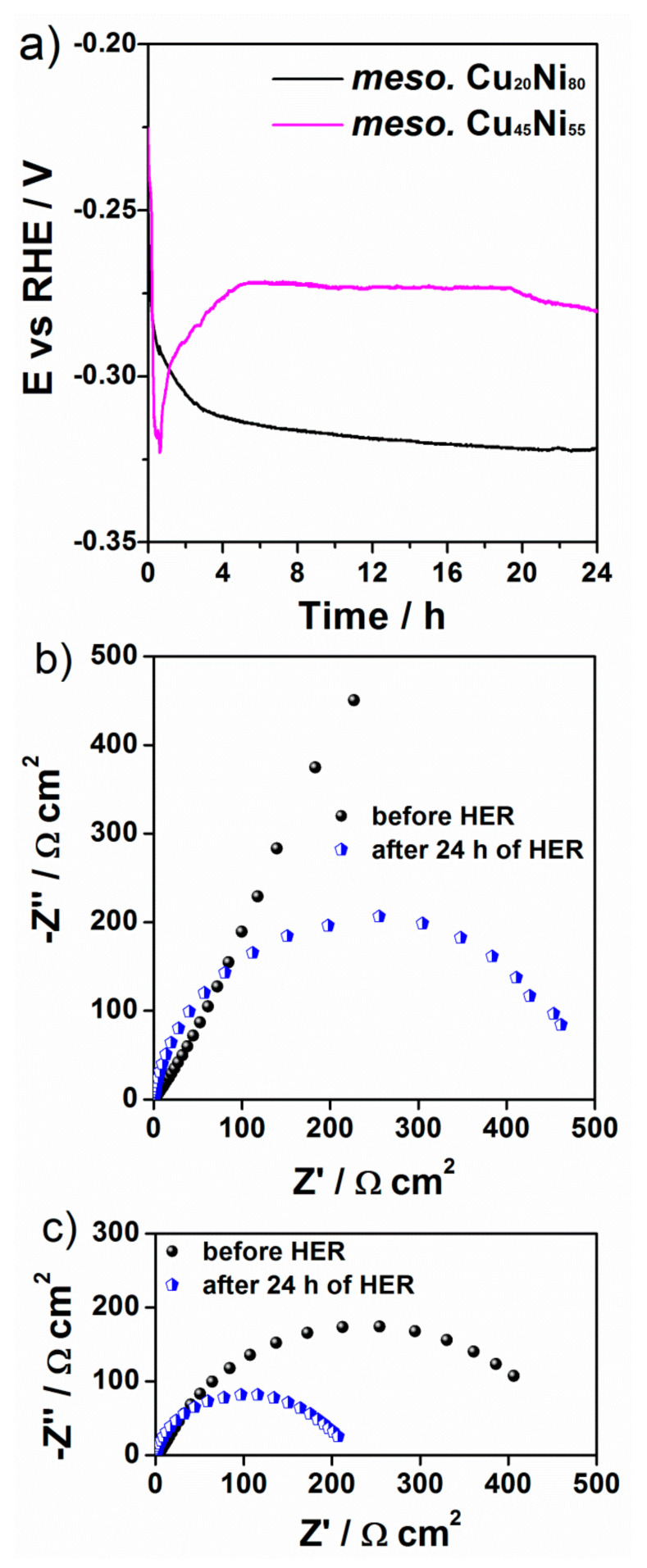
Potential as a function of time during galvanostatic HER at −10 mA·cm^−2^ for surface-mesostructured CuNi films (**a**). Nyquist plots before and after 24 h durability tests for surface-mesostructured films with composition (**b**) Cu_20_Ni_80_ and (**c**) Cu_45_Ni_55_.

**Figure 11:**
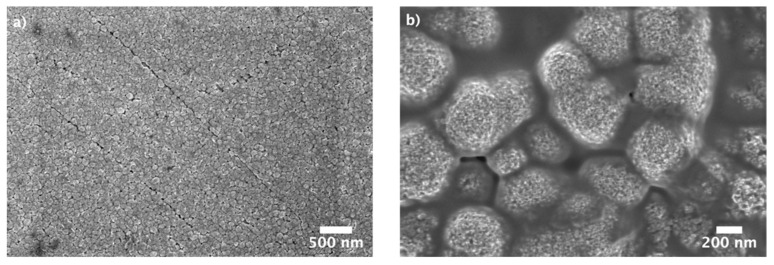
SEM images of surface-mesostructured films with composition (**a**) Cu_20_Ni_80_ and (**b**) Cu_45_Ni_55_ after 24 h durability tests.

To further investigate the stability of the CuNi catalysts, the potential was recorded for 24 h at an applied geometric current density of −10 mA·cm^−2^ (Figure 10a). For the Cu_20_Ni_80_ film, the potential dropped from −260 mV to −320 mV during the first 2 h; then the change in potential slowed down, and, from 6 h onward, little or no variation was detected (i.e., the potential became stable). The potential also shifted toward more negative potential for the Cu_45_Ni_55_ film during the first 45 min but then it partially recovered and became stable from 5 h until the end of the experiment (E = −270 mV). These results suggest that the CuNi films experienced a decrease in the catalytic activity during the first hours, but no further decline of the activity was noticed after 5–6 h of continuous galvanostatic HER. Figure 10b,c show the Nyquist plots before and after the 24 h durability test. For both compositions of surface-mesostructured CuNi films, the impedance decreased, as indicated by smaller semicircles, hinting at an electrochemical activation of both surfaces. Finally, the morphology of the CuNi catalysts after being subject to 24 h HER experiment was investigated by SEM (Figure 11). No pronounced changes of film morphology were observed, although the surfaces seemed to be partially covered by an insulating thin layer, which could be partly attributed to remnants of KOH.

The surface-mesostructured CuNi materials were comparable to or slightly outperformed other Ni-based catalysts reported in the literature for HER in alkaline media [39], but were below the performance of noble-metal-based catalysts such as Ru and Pt with dissimilar architectures [40]. For example, Ru nanoparticles dispersed within a nitrogenated holey two-dimensional carbon structure exhibited an overpotential of 17 mV at −10 mA·cm^−2^ in 1.0 M KOH solution [41]. Although the performance of the here-deposited non-noble-metal-based films cannot yet compete against noble-metal catalysts, the values could be brought closer with further optimization of the catalyst surface roughness and composition.

## 4. Conclusions

In summary, the HER activity of electrodeposited bimetallic CuNi films was investigated in alkaline media, and the morphological and structural features of the samples were correlated with their electrocatalytic behavior. Interestingly, the surface topology and composition of the CuNi layers can be controlled by changing the pH value of the electrolyte, from Cu_20_Ni_80_ at quasi-neutral pH to Cu_45_Ni_55_ at pH = 4. The surface-mesostructured Cu_45_Ni_55_ films exhibited lower onset for HER compared to plain Ni and Cu_20_Ni_80_ films. Moreover, the surface-mesostructured Cu_45_Ni_55_ film showed higher specific activity than Cu_20_Ni_80_ in spite of its lower Ni/Cu ratio. The reason for this previously considered counterintuitive result was correlated to an increase in the surface Ni active sites, as demonstrated by cyclic voltammetry studies.

## Figures and Tables

**Table 1:** ECSA, overpotential at −10 mA·cm^−^^2^ (η_10_), Tafel slope (*b*), and TOF values for the deposited Cu_20_Ni_80_ and Cu_45_Ni_55_ surface-mesostructured films. The ECSA values are given for Cu and Ni elements separately. Values in parentheses correspond to plain films of same composition.

	Cu_20_Ni_80_	Cu_45_Ni_55_
ECSA (Cu)	0.3 ± 0.05 m^2^·g^−^^1^	0.4 ± 0.1 m^2^·g^−^^1^
ECSA (Ni)	7.4 ± 0.4 m^2^·g^−^^1^	12.5 ± 0.3 m^2^·g^−^^1^
η_10_	210 (220) mV	210 (230) mV
*b*	65 (115) mV	68 (97) mV
TOF	0.5 s^−^^1^	0.3 s^−^^1^

## Data Availability

The data presented in this study are available on request from the corresponding author.
